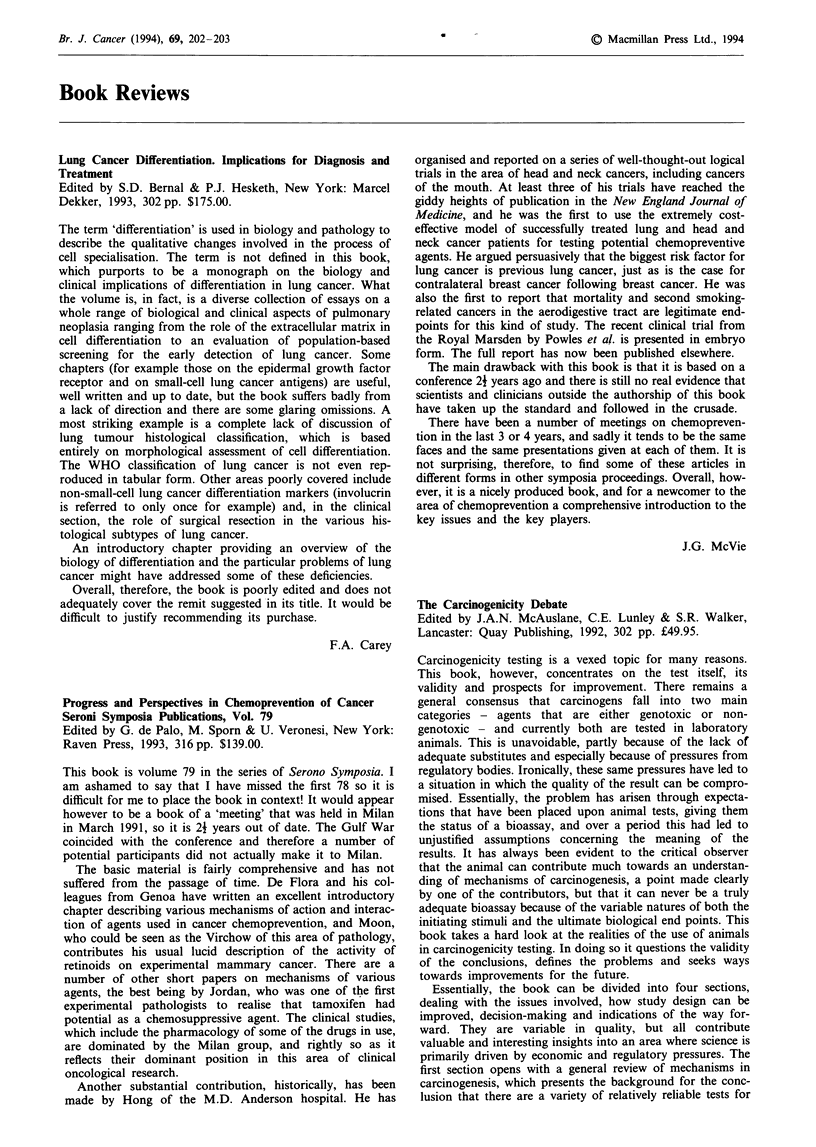# Progress and perspectives in chemoprevention of cancer seroni symposia publications, Vol. 79

**Published:** 1994-01

**Authors:** J.G. McVie


**Progress and Perspectives in Chemoprevention of Cancer Seroni Symposia Publications, Vol. 79**
Edited by G. de Palo, M. Sporn & U. Veronesi, New York: Raven Press, 1993, 316 pp. $139.00.

This book is volume 79 in the series of *Serono Symposia*. I am ashamed to say that I have missed the first 78 so it is difficult for me to place the book in context! It would appear however to be a book of a 'meeting' that was held in Milan in March 1991, so it is 2½ years out of date. The Gulf War coincided with the conference and therefore a number of potential participants did not actually make it to Milan.

The basic material is fairly comprehensive and has not suffered from the passage of time. De Flora and his colleagues from Genoa have written an excellent introductory chapter describing various mechanisms of action and interaction of agents used in cancer chemoprevention, and Moon, who could be seen as the Virchow of this area of pathology, contributes his usual lucid description of the activity of retinoids on experimental mammary cancer. There are a number of other short papers on mechanisms of various agents, the best being by Jordan, who was one of the first experimental pathologists to realise that tamoxifen had potential as a chemosuppressive agent. The clinical studies, which include the pharmacology of some of the drugs in use, are dominated by the Milan group, and rightly so as it reflects their dominant position in this area of clinical oncological research.

Another substantial contribution, historically, has been made by Hong of the M.D. Anderson hospital. He has organised and reported on a series of well-thought-out logical trials in the area of head and neck cancers, including cancers of the mouth. At least three of his trials have reached the giddy heights of publication in the *New England Journal of Medicine*, and he was the first to use the extremely cost-effective model of successfully treated lung and head and neck cancer patients for testing potential chemopreventive agents. He argued persuasively that the biggest risk factor for lung cancer is previous lung cancer, just as is the case for contralateral breast cancer following breast cancer. He was also the first to report that mortality and second smoking-related cancers in the aerodigestive tract are legitimate end-points for this kind of study. The recent clinical trial from the Royal Marsden by Powles *et al.* is presented in embryo form. The full report has now been published elsewhere.

The main drawback with this book is that it is based on a conference 2½ years ago and there is still no real evidence that scientists and clinicians outside the authorship of this book have taken up the standard and followed in the crusade.

There have been a number of meetings on chemoprevention in the last 3 or 4 years, and sadly it tends to be the same faces and the same presentations given at each of them. It is not surprising, therefore, to find some of these articles in different forms in other symposia proceedings. Overall, however, it is a nicely produced book, and for a newcomer to the area of chemoprevention a comprehensive introduction to the key issues and the key players.

J.G. McVie